# Product News

**DOI:** 10.1155/S1463924681000126

**Published:** 1981


### Product News

**Editor's Note:** *The address of the manufacturer/supplier appears in italics at the end of each item. In some cases this address will be that of a subsidiary to the manufacturing company as the address given is that from which the information has been obtained.*

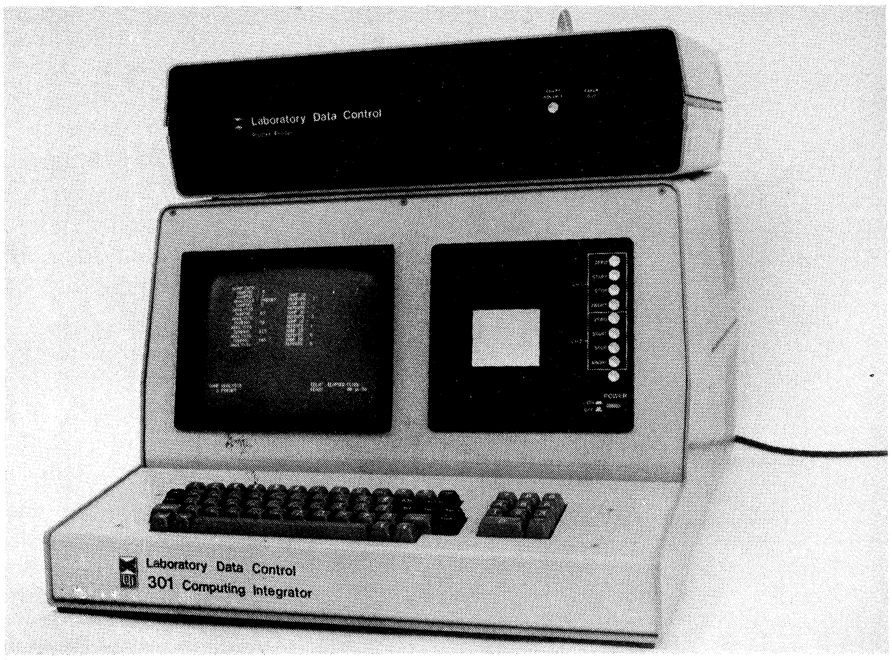

*Computing integrator system from Laboratory Data Control*

### Computing integrator

Laboratory Data Control have announced the introduction of a computing integrator, the 301 system, which can be supplied as a dual system or a single channel integrator to be upgraded to a dual channel system at a later date. The 301 system consists of two channels of data acquisition, 16K bytes of storage memory (expandable to 32K bytes), an inbuilt cassette recorder and an interactive visual display. Programming is via a full 64-character keyboard or by tape input. The system outputs to a printer plotter and/or via an RS 232 port. The printer plotter can be either a single or a two-pen recorder. The plotter provides plotting of the output of two detectors, printout of data report on the same chart as the chromatogram, annotation of the peak retention times and hardcopy of all chromatographic parameters. During a run both chromatograms can be stores, one in core and one on tape for later reprocessing. Reprocessing of a stored chromatogram includes the ability to optimise peak separation by changing 'N' times the type of base-line handling parameters, replotting of the stored chromatograms and the ability to change all post-run calculation parameters. An important feature of this microprocessor-based system is the ability to access all data files (and a number of other files) by BASIC for unlimited calculations.

*Laboratory Data Control, Building 89, Industrial Estate, Shannon Airport, Co. Clare, Ireland.*

### GC system

The Vista 44 gas chromatography system from Varian has all-digital parameter controls that can be programmed remotely from the connected terminal. It has a large column oven and can be equipped with any one of six detectors for high resolution chromatography. The system is expandable to include three additional Vista GC's and/or LC's or it will handle data from any three LC's and/or GC's. The Vista 44 uses the same columns as the Varian GC 3700 series and can also be equipped with Varian AutoSamplers. The integrated Vista 401 data system provides sophisticated handling and automatic instrument control for up to four chromatographs in a wide variety of configurations. Key features of the data system are a powerful interactive CRT/keyboard (real time CRT status display), floppy disk storage with the possibility of storing complete chromatograms, a fixed head dual printer/plotter for one-source chromatograms and reports, interface capability with both large and small computers, and pre-programmed methods for most standard calculations.

*Varian AG, Steinhauserstrasse, CH-6300, Zug, Switzerland.*

### Additonal chemistries

Following the introduction of Astra, Beckman's automated chemistry analyser, six additional chemistries have been added to the programme to extend the system's capabilities. The original chemistries available are glucose, BUN, creatinine, chloride, sodium and potassium. Now total protein has been added together with cerebrospinal fluid total protein, albumin, amylase, calcium and total bilirubin.

*Becknan-RIIC Ltd, Turnpike Road, Cressex Industrial Estate, High Wycombe, Bucks, UK.*

### pH meter

A microprocessor-controlled pH meter designated PHM83 Autocal has been introduced by Radiometer for routine measurements of pH or mV and temperature. The instrument has one-key autocalibration without switches or dials and automatic recognition of specified buffer in any order. The correct buffer value at the actual measuring temperature is stored in the memory of the microprocessor. The 20-character alphanumeric display informs the operator of the calibration progress, electronic stability and possible operator errors or electrode failures. A test program allows the operator to check the status of the pH meter and a built-in service program continuously checks the microprocessor. A permanent memory retains all calibration data during power off.

*Radiometer A/S, Emdrupvej 72, DK-2400, Copenhagen NV, Denmark.*

### ICP source unit

The latest version of the Philips inductively coupled plasma source unit is said to offer improved performance for the analysis of aqueous and organic solutions by emission spectrometry. Based on the work of Dr Boumans and his team in the Philips research laboratories, the PV 8490 source can be fitted to the company's range of air-path and vacuum spectrometers. This ICP system now incorporates a torch with revised geometry designed to give more efficient plasma generation. A slight extension of the outer quartz tube above the work coil enhances stability without inhibiting measurement close to the coil in cases where this offers the best observation zone. Modifications to the intermediate tube and sample injection also overcome the problems of salt deposits from aqueous solutions and carbon build-up from organic materials. A choice of two nebulisers is available: a standard pneumatic crossflow type constructed in glass and an optional Teflon capillary model. The latter can be used for special applications such as the analysis of highly corrosive solutions.

*NV Philips' Gloeilampenfabrieken, PO Box 523, 5600 AM Eindhoven, The Netherlands.*

### Viscometer with automatic density compensation

In the Nametre Model 7.010 vibrating sphere viscometer density compensation is obtained by a ten-turn counting dial that can be locked in the set position. The standard dial can be set from 0.400 to 1.400 density values. By dialling any density between the above values the electronic control unit will automatically make the division and display the result in centipoise or centistoke units. Should the compensator dial be accidentally offset from the 1.000 position by ± 0.1 turn then a red warning light bar marked "DENSITY" on the display will indicate that the density compensation computer circuit has been activated. The viscometer displays viscosity in six linearised bands in the range $10^{-1}$ to $10^5$ cp.

*Nametre Company, 1778 State Highway 27, Edison, NJ 08817, USA.*

### Multi-balance interfacing

A programmable multi-balance interface unit from Telmoss connects up to five electronic balances to a computer or display/printer terminals using two RS232C or 20 mA current loop outlets. The unit is microprocessor based and is compatible with balances from the Sartorius MP range and with the Mettler PL or PK series. The balances can be of several types or all the same. This unit is applicable for production and quality control in any industry which monitors weights, either on intermediates or on finished products. It has the flexibility to allow many types of systems to be assembled with programs tailored to individual requirements. A number of the interface units can be connected either in radial or daisy-chain configuration.

*Telmoss Ltd, 28 Dellcott Close, Welwyn Garden City, Herts, UK.*

### Automatic dissolution monitoring

The versatility of the Pye Unicam SP-100/150 series spectrophotometers has been extended by the introduction of an automatic six cell dissolution accessory. The system conforms to all current regulatory procedures in the pharmaceutical industry. It allows discrete monitoring of up to six samples against a standard or reference. Measurement intervals of 30 seconds to 99 minutes can be conveniently selected and, if required, multi-wavelength measurements and superimposed traces can be obtained on the built-in recorder. The addition of an HP97S programmable calculator allows presentation of analytical results directly in concentration with sample number. Alternatively, on input of dissolution volume, results will be printed as weight of active component dissolved. Although designed for the pharmaceutical industry and tablet dissolution applications in mind, the system is readily adaptable to any dissolution requirement; e.g. confectionery, food, agricultral or chemical industries in both quality control and research applications.

*Pye Univam Ltd, York Street, Cambridge, CB1 2PX, UK.*

### Luminescence photometer

The Turner Designs Model 20 luminescence photometer from Techmation is a microprocessor-controlled instrument for both bio and chemi-luminescence analyses. Typical applications include quantification of adenosine triphosphate for biomass determinations in marine and fresh water, control of activated sewage sludge and determination of cell viabilities in a variety of environmental, agricultural and clinical fields. The Model 20 uses a through-period optical system which includes a stabilised light source and computer-controlled shutter. This gives a system which is not only stable but also requires the minimum of day-to-day adjustments. To protect the photomultiplier from light overload the shutter closes within 50 milliseconds of light entering the instrument. This feature also simplifies sample or reagent introduction procedures. Fully selectable delay and assay times allow measurements by either peak height or integrate methods.

*Techmation Ltd, 58 Edgware Way, Edgware, Middx, HA8 8JP, UK.*

### Notes for Contributors

### Presentation of manuscripts

Manuscripts should be typed (double-spaced) on one side of the paper only and with generous margins. The title should be brief and informative avoiding the word "new" and its synonyms. The full list of authors with their affiliations and full address(es) should appear on the title page. On a separate sheet an abstract of no more than 150 words is required. This should succinctly describe the scope of the contribution and highlight significant findings or innovations. It should be written in a style which can easily be translated into French and German.

*The Concise Oxford Dictionary* and Fowler's *Modern English Usage* (both published by Oxford University Press) should be used as the standard for spelling and grammar. Abbreviations should be limited to those generally recognised, or where a frequently occuring term is abbreviated it should, in the first instance, be explained thus "flow injection analysis (FIA) ..." and the abbreviation used thereafter. Abbreviations, for standard measures and units should follow SI recommendations. There are various publications giving guidance on the use of SI units.

References should be indicated in the text by numerals following the author's name, i.e. Skeggs [6]. On a separate sheet of paper, list all references in numerical order thus:

[6] Skeggs, L.T., *American Journal of Clinical Pathology*, 1959, **28**, 311.

Note that journal titles are given in full. Where there is more than one author, the form Foreman et al. should be used in the text but all authors should be named in the list of references. When reference is made to a chapter in a book the reference should take the following form:

[7] Malmstadt, H.V. in "Topics in Automatic Chemistry" Ed. Stockwell P.B. and Foreman J.K. 1978 Horwood, Chichester, pp. 68-70.

Only work which has been published or has been accepted for publication should be cited. Avoid the citation of documents which are subject to restricted circulation, patent literature, unpublished work and personal communications. The latter can be mentioned in the text in parenthesis.

To illustrate a paper line diagrams are preferred to photographs. Photographs should only be used when they significantly add to the discussion. Diagrams, charts and graphs should be carefully drawn in black ink on stout card or heavy quality tracing paper. Most illustrations are reduced for publication; to allow for this originals should be between 16 and 36 cm wide (the depth must not exceed 50 cm). The lettering of diagrams should be sufficiently clear to withstand reduction. Except in the case of proper names, all lettering should be in lower case print. If photographs are used they must be supplied in the form of clear, unmounted, glossy, black and white prints. "Instant" photographs are not normally acceptable. All illustrations must be identified on the reverse showing the figure number and the author's name.

Each illustration should have a fully explanitory caption. Captions should be typed together on a separate sheet of paper; they must *not* be an inseparable part of the illustration.

